# Training environment and sponsorship dynamics in Hungarian artistic swimming

**DOI:** 10.3389/fspor.2024.1481661

**Published:** 2024-10-11

**Authors:** Domicián Máté, Jolita Vveinhardt, Anna Fekete

**Affiliations:** ^1^ Department of Engineering Management and Enterprise, Faculty of Engineering, University of Debrecen, Debrecen, Hungary; ^2^ DHET-NRF Sarchi Entrepreneurship Education, Department of Business Management, University of Johannesburg, Johannesburg, South Africa; ^3^ Institute of Sport Science and Innovations, Lithuanian Sports University, Kaunas, Lithuania; ^4^ Faculty of Economics and Social Sciences, Centre for Physical Education, Budapest University of Technology and Economics, Budapest, Hungary

**Keywords:** artistic swimming, training environment, funding, sponsorship, aquatic sports infrastructure

## Abstract

The present study examines the training environment and sponsorship dynamics in Hungarian artistic swimming, with a particular focus on competitiveness. Through the utilization of a linear regression method ample aspects were identified, such as coach qualifications, funding sources, and facilities, that positively impact the number of qualified competitors. The availability of funding sources, when coupled with governmental assistance, has a favorable effect on the number of competitors, whereas membership fees have a deleterious impact. Furthermore, the findings indicate that increased water availability and increased publicity enhance artistic swimming recognition, whereas improved land-based training conditions may lead to a decline in the number of competitors. This paper discusses how different aspects of training programs can be integrated to improve overall athleticism, prevent injury, and enhance the competitive ability of artistic swimmers.

## 1 Introduction

Artistic swimming, previously designated synchronized swimming, is an Olympic sport comprising the following categories: solo (for both gender competitors), duet, mixed duet (the inaugural Olympic event in this category will take place in Paris 2024), team, and combined free and highlight. Artistic swimming is widely regarded as an aesthetic sport, with athletes’ abilities being evaluated by a panel of judges according to subjective criteria (1). The current evaluation criteria were established by the Fédération Internationale de Natation (FINA) and are applicable for the period between 2022 and 2025 (2).

The multifaceted nature of synchronized swimming is demonstrated by the need for physical and mental strength, improved cardio-respiratory and metabolic capacities, flexibility, a sense of rhythm, and an appreciation of artistic nuances (3). For athletes to be adequately prepared, it is essential that their training incorporates both land-based and aquatic exercises (4). The former encompasses mobility training, breathing techniques, counting exercises, and conditioning training. In contrast, the latter involves technical training, routine practice sessions, and swimming. Therefore, there should be a plethora of accessible materials, resources, and facilities to facilitate the mastery of each area related to artistic swimming (5).

There has been a notable surge in the publication of research articles about artistic or synchronized swimming. The most extensively studied areas within this field are physiology, sports performance, and injuries (6). While health sciences and medicinal disciplines continue to attract the attention of researchers (7), there has been a discernible rise in interest in the social and economic implications of artistic swimming.

The psychological factors that influence participation in sports have also been the subject of previous research. The decision of whether to allow children to participate in sports is largely dependent on the values of their parents with respect to the practice of sports in general and their socioeconomic status. In families where parents prioritize participation in sports and are willing to adjust their family life around the children's training schedules, children are more likely to engage in sports (8). The research findings show that involvement in artistic swimming requires substantial and enduring financial investment from parents. Furthermore, these findings suggest that children engaged in artistic swimming typically have parents with higher annual incomes than do children involved in nonaquatic sports such as athletics. Another viewpoint to consider is that of coaching styles and their influence. Athletes, especially those at the elite level, spend a significant amount of time with their coaches. As a result, the training and communication approaches of coaches can completely influence behavior, enjoyment, and, ultimately, the commitment of athletes to the sport (9).

The 2015 Sport Development Concept (SDC) of the Hungarian Synchronized Swimming Federation established a series of objectives with the aim of stimulating the advancement of artistic swimming (10). The concept of sport development provided a comprehensive guide to areas of artistic swimming in need of improvement, with a focus on the goals of developing member clubs and how the age, gender or qualifications of coaches influenced the participants in the study. One of the most pressing issues for the development of artistic swimming in Hungary is the training of coaches. According to the SDC, it is recommended that the governing body collectively decide that within the next three years, each club should have at least one coach with a bachelor's degree (BSc) (intermediate coach), and within the next five years, at least one coach with a master's degree (MSc) (professional coach). It is also important to ensure that only individuals with at least an OKJ registered qualification (vocational coach) are allowed to participate in coaching.

Nevertheless, there is a need for more discourse on the issues that have the potential to influence the number and performance of artistic swimmers. The primary goal of this study is to investigate the factors that affect competitive participation in artistic swimming in Hungary. This topic focuses on the administrative sides of artistic swimming and discusses the role of sports management in creating competitive opportunities, managing facilities, and organizing events. The study concentrates on the specific areas of training environments and sponsorship dynamics. It aims to analyze participant demographics and evaluate coaching training opportunities within the country. Ultimately, the study aims to provide insights that can help improve the growth and sustainability of artistic swimming in Hungary. The importance of this study lies in its attention to the deficiency of media coverage and the limited sports development opportunities for artistic swimmers, and training conditions. Artistic swimming is a minority sport with limited economic power; resources and research on the subject are scarce in Hungary.

This research seeks to address the following questions: (1) What strategies can be employed to increase the number of certified competitors? (2) Do the quality of the infrastructure and the nature of the revenues impact the number of riders? (3) Are the current training conditions suitable for coaches and athletes to develop within the sport, from both coaching and competitive perspectives? (4) What are the primary factors that influence the competitiveness of Hungarian artistic swimming clubs, particularly in terms of the number of certified competitors?

## 2 Materials and methodologies

The statistical sampling was based on the number of 21 Hungarian member clubs registered under the Hungarian Synchronized Swimming Federation (MSZUSZ) in 2023 (11). These data were selected as the basis for the sampling due to their representativeness of the larger population of Hungarian clubs within the wider context of Hungarian artistic swimming. The data presented in this research are derived from the analysis of responses to an anonymous and voluntary questionnaire comprising 28 questions.

After all the clubs were reached, only a small number responded within the designated timeframe. This occurred because the training schedule had not been finalized after the summer break. However, 20 or more measurements are needed to achieve a confidence level of 95% such that the real value is within ±5% of the measured value. The collected sample size of 16 is sufficiently large to provide reliable data, minimizing the likelihood of errors. This population represents 76.19% of the total population, suggesting that the responses are likely to be representative of the broader population.

The survey questions were designed and sent out on the basis of the opinions of two randomly selected CEOs of member clubs. The inquiry was conducted in accordance with established ethical protocols, including online, telephone, and face-to-face interrogations.

The studies on people and synchronous clubs were carried out with the approval of the MSZUSZ. The trials were conducted in accordance with local legislation and institutional requirements. Written consent from competitors or legal relatives was not required for participation, as the collection procedures were not conducted as part of training sessions led by the coaches.

### 2.1 Participants

A fundamental problem in artistic swimming is the “aging” of athletes. Competitors leave the sport at a relatively early age because of their studies or other commitments (12). In 43.75% of the clubs surveyed, both the children's and junior age groups dominated, with high numbers of club members. In comparison, 25 percent have the highest number of junior age group members, and 18.75 percent have the highest number of adult age group members. The remaining 12.5% are split between clubs with predominantly children and junior age groups. In general, the number of club members tends to decrease with age. This phenomenon has a significant effect on the competitive landscape of artistic swimming clubs, particularly in terms of the number of certified competitors.

This can be seen not only at the level of individual clubs but also at the national level. At the national level, the average number of members in the children's and junior age groups is 11–15, whereas the junior and adult age groups have 6–10 members. None of the clubs surveyed have para-synchronized swimmers. Currently, a quarter of the Hungarian clubs surveyed have male swimmers, typically between 1 and 5. Compared with that of female participants, the number of male competitors is minimal, with a maximum of 20 at the national level.

One of the pertinent SDC objectives is to eliminate the presence of unqualified coaches in clubs (10). However, the conceptualization of coaching remains undetermined, but most associations continue to align with the projected benchmarks for the ratio of professional to intermediate coaches. This goalmouth has been achieved for 56.25% of the respondents, whereas the remaining 43.75% continue to employ unqualified coaches. Among the 16 respondents, 14 indicated that their association has at least one professional coach, and one indicated that it has more than five. A total of two respondents indicated that their respective association did not employ a coach. A total of 68.75% of the respondents indicated that their association employs at least one professional coach with an intermediate qualification.

### 2.2 Design of the survey

The design of the survey was influenced by the subsequent inquiries, which facilitated the delineation of the areas requiring examination. The dependent variable employed in the models was the number of certified competitors, which was in accordance with the question “How many certified competitors does your club/section have?” was included in the questionnaire. For the explanatory variables, a Likert scale (1–5) was also employed for the number of coaches (0–5), the number of sponsorships and the responses provided by coaches and owners.

The descriptive statistics (e.g., mean, standard deviation, minimum and maximum values) and labels for each variable are summarized in the following tables (Tables 1, 2).

**Table 1:** Summary of variable labels and descriptions.

Variables	Abbreviations	Description
Dependent variable		
Number of certified competitors	COMP	How many certified competitors does your club/section have?
Explanatory variables		
Number of coaches	PCOA	How many professional coaches assist the club/section of the club?
	SCOA	How many secondary-level coaches assist the club/section of the club?
	QCO	How many qualified (OKJ) coaches assist the club/section of the club?
	UCOAC	How many unqualified coaches assist the club/section of the club?
	CCOA	How many competitor coaches assist the club/section of the club?
Sources	FEES	How much do the membership fees contribute to the income of the association?
	SPON	How much do the sponsors contribute to the income of the association?
	MUNI	How much do the municipal subsidies contribute to the income of the association?
	SAID	How much does the state aid contribute to the income of the association?
	OSOU	How much do other sources contribute to the income of the association?
Training conditions	CSUL	Do you think that the conditions provided by the sports facility are suitable for training on land?
	CSUA	Do you think the conditions provided by the sports facility are suitable for aquatic training?
	ETAM	Do you think the current amount of training is enough to prepare you?
	ETLT	Do you think the quantity/quality of equipment available for land-based training is adequate?
	ETWT	Do you think the water surface available for water training is adequate?
	EPUB	Do you think artistic swimming as a sport gets enough publicity?

Authors’ compilation.

**Table 2:** Descriptive statistics of the variables (*N* = 16).

Variables	Abbreviations	Mean	Standard Deviations	Min	Max
Dependent variable					
Number of certified riders	COMP	4.188	1.109	2	5
Explanatory variables					
Number of coaches	PCOA	1	0.632	0	3
	SCOA	0.875	0.718	0	2
	QCOA	1.188	0.655	0	3
	UCOA	0.5625	0.727	0	2
	CCOA	1.313	1.448	0	4
Sources	FEES	4.438	0.813	2	5
	SPON	1.813	0.910	1	4
	MUNI	1.5	0.730	1	3
	SAID	1.375	0.619	1	3
	OSOU	1.5	0.730	1	3
Training conditions	CSUL	3.125	1.258	1	5
	CSUA	4	0.730	2	5
	ETAM	3.313	1.195	2	5
	ETLT	2.625	1.147	1	5
	ETWT	3.125	1.31	1	5
	EPUB	1.75	0.774	1	4

### 2.3 Statistical analysis

Ordinary Least Squares (OLS) regression is commonly used to approximate multivariate statistical models. The regression analysis was performed via the latest (2024) release of Gretl (13), a cross-platform software package tool for econometric analysis. Multivariate linear regression is a statistical method that enables the calculation of a continuous dependent variable (e.g., the number of certified riders) can be predicted from a set of independent variables. Moreover, it facilitates the assessment of the extent to which the explanatory (regressor) variables, including the quantity and quality of coaches, financial aid, and club environment account for the variability in the dependent variable.

The following formula, represented in matrix form (Equation 1), was utilized:

y=Xβ+e(1)

In the context of linear regression, the notation (y) represents the *n* × 1 vector of output observations, (X) denotes the *n* × (p + 1) matrix of values of explanatory variables (including the constant), and (e) signifies the *n* × 1 vector of errors (14).

When testing the hypotheses, we utilized linear parameters and conducted OLS regression analyses with heteroskedasticity-consistent (HAC) and robust standard errors. The results from these regression models indicate a lack of heteroskedasticity, with the disturbances exhibiting consistent variance across all observations.

The models include one dependent variable and three independent (explanatory) variables for the Hungarian case (i). In Equation (2), we assume (H1) that *the number of qualified coaches positively affects the number of certified competitors*:

$$\begin{array}{rl} \mathrm{COM}P_{i} & =\;\beta_{0}+\;\beta_{1}\;\mathrm{PCO}A_{i}+\beta_{2}\;\mathrm{SCO}A_{i}+\beta_{3}\;\mathrm{QCO}A_{i}\\ & \;+\beta_{4}\;\mathrm{UCO}A_{i}+\beta_{5}\;\mathrm{CCO}A_{i}+e_{i} \end{array}\;\left(2\right)$$

where COMP is the number of certified competitors and professional (PCOA), secondary (SCOAC), qualified (QCOA), unqualified (UCOA) and competitor (CCOA) coaches. In Equation (2), it is assumed (H1) that *club resources influence the number of certified drivers:*

$$\begin{array}{rl} {\mathrm{COMP}}_{i} & =\;\beta_{0}+\;\beta_{1}\;{\mathrm{FEES}}_{i}+\beta_{2}\;{\mathrm{SPON}}_{i}+\beta_{3}{\mathrm{MUNI}}_{\text{i}}\\ & \;+\beta_{4}\;{\mathrm{SAID}}_{i}+\beta_{5}\;{\mathrm{OSOU}}_{i}+e_{i} \end{array}\;\left(3\right)$$

where (FEES) denotes the contribution of membership fees, (SPON) denotes sponsors, (MUNI) denotes municipal subsidies, and (SAID) denotes state aids and other resources (OSOU). In Equation 3 (H2), it was assumed that *the various subsidies and revenues available to the club have an impact on the number of certified riders* from the perspective of trainers and owners:

$$\begin{array}{rl} \mathrm{COM}P_{i} & =\;\beta_{o}+\;\beta_{1}\;\mathrm{CSU}L_{i}+\beta_{2}\;\mathrm{CSU}A_{i}+\beta_{3}\mathrm{ETA}M_{i}\\ & \;+\beta_{4}\;\mathrm{ETL}T_{i}+\beta_{5}\;\mathrm{ETWT}+e_{i} \end{array}\;\left(4\right)$$

where (CSUL) and (CSUA) denote the land and water training conditions, respectively; (ETAM) represents the amount of training; (ETLT) represents the quantity and quality of land equipment; and (ETWT) represents the water surface and publicity (EPUB). In Equation (4) (H3), it was assumed that *the training conditions in the club had a positive effect on the number of certified competitors*.

All explanatory variables were subsequently tested and then individually deleted from the full model via two-sided p values (<0.1) until all remaining variables contributed significantly to the model. The R-squared values, also referred to as coefficients of determination, serve as a metric for evaluating the degree of fit exhibited by a linear regression model in relation to a given dataset. The variance inflation factor (VIF) values indicate that multicollinearity is not a significant problem. In all cases, the VIF values are below the maximum acceptable level of ten (15) or even less than 5 (16). The Doornik-Hansen (DH) tests indicate the presence of multivariate normality of the residuals at a significance level of 0.05 (5%) (17).

## 3 Results

### 3.1 Analyzing appropriate training conditions

The nature of the research methodology made it possible to study the duration, frequency, equipment, and external conditions of training sessions and to gather the opinions of coaches on the subject. Importantly, the studies carried out in this area are not strictly related to the objectives but aim to assess the training conditions in swimming sports.

81.25 percent of the clubs have the possibility to organize land-based training sessions year round, whereas the remaining 18.72 percent can do so only intermittently. The corresponding figure for aquatic sessions is 93.75 percent, with only 6.25 percent of respondents indicating that such sessions are held only on an intermittent basis. The mean duration of dryland training sessions is one hour, irrespective of age. However, the number of sessions decreases with age. On average, children, juniors, and youth have three dryland training sessions per week, whereas adults have two.

The mean duration of aquatic training sessions for children is 1.28 h, with an average of 3.25 sessions per week. For the junior-aged group, the mean duration of aquatic training sessions was 1.47–3.32 h, whereas for the adult-aged group, it was 0.93 per hour, for a total of −2.5 sessions. These findings suggest that there is a significant decrease in training amount with age, which appears to be accurate. Furthermore, the amount of training increases after the age of 12, with the junior age group engaging in the most training, both in terms of duration and frequency. However, this trend gradually diminishes as age increases.

The coaches were requested to provide a rating of their opinions on the infrastructure and the quantity of water surface and equipment available for training on a 5-point Likert scale. A total of 56 percent of the respondents indicated a degree of satisfaction with the conditions provided by the on-land sports facility. Moreover, this figure increased to 87.5% for the aquatic facility. Nevertheless, 56.25% of the respondents considered the quantity of facilities and water surface area to be inadequate and the quality of the facilities on land to be unsatisfactory.

### 3.2 Results of the regression analysis on certified competitors, quality of coaches, subsidies and club conditions

Three regression models were estimated via equations (1), (2) and (3). The regression coefficients in Table 3 shows that professional coaches (0.966), trainers with intermediate qualifications (0.727), unqualified (0.562) and competitor coaches (0.250) exhibit distinct patterns. Incorporating this group is warranted, as it serves as a substantial predictor of the number of confirmed participants. The H1 hypothesis can be accepted to a certain extent in that the number of coaches and their qualifications have a positive effect on the qualified competitors, except for those with OKJ qualifications.

**Table 3:** OLS regression model estimates based on Equation 1 (*N* = 16).

Dependent variable	COMP					
Explanatory variables	Model 1	Model 2	Model 3	Model 4	Model 5	Model 6
Constant	3.265	3.544	3.512	3.875	4.060	2.735
	(6.07)***	(8.64)***	(6.08)***	(11.44)**	(7.61)***	(6.88)***
PCOA	0.966					0.957
	(1.95)*					(2.37)**
SCOA		0.727				
		(3.54)***				
QCOA			0.675			
			(1.72)			
UCOA				0.562		
				(3.29)***		
CCOA					0.155	0.250
					(1.09)	(2.23)**
Adj. *R*^2^	0.158	0.434	0.115	0.397	0.013	0.530
*R* ^2^	0.214	0.472	0.174	0.437	0.078	0.593
F value	3.823*	12.537***	2.960	10.884***	1.198	9.487***
max (VIF)	–	–	–	–	–	1.022
DH-test	4.662*	0.947	3.013	3.378	6.877*	2.188
Observations	16					

Heteroskedasticity‒robustness (HAC) *t* statistics for standard errors are given in parentheses.

*** Significant at the 0.001 level (*p* < 0.001), **0.05 *p*-level, *0.1 *p*-level.

Furthermore, coefficients were calculated for the dimensions of organisational support, as presented in Table 4. The models, except for state aid (see Model 4), demonstrate a significant effect on the influence of the subsidies. The H2 hypothesis can be partially accepted, namely, that the number of confirmed competitors is significantly influenced by individual sources of funding, apart from state support. Moreover, it is unsurprising that remuneration paid to competitors (−0.334) in the form of membership fees has a negative effect on the number of competitors. The imposition of such fees is probably attributed to the financial strain they impose on participants. The number of confirmed participants is subsequently positively influenced by sponsors (0.484) and municipal support (0.510), as well as other forms of support (0.500).

**Table 4:** OLS regression model estimates based on Equation 2 (*N* = 16).

Dependent variable	COMP					
Explanatory variables	Model 1	Model 2	Model 3	Model 4	Model 5	Model 6
Constant	6.294	3.574	3.455	3.613	3.489	4.426
	(4.66)***	(7.43)***	(6.80)***	(6.42)***	(7.82)***	(7.06)***
FEES	−0.483					−0.334
	(−1.62)					(−2.83)**
SPON		0.484				0.515
		(2.48)**				(2.73)**
MUNI			0.510			0.231
			(2.59)**			(2.28)**
SAID				0.434		
				(1.50)		
OSOU					0.500	
					(2.58)**	
Adj. *R*^2^	0.097	0.256	0.276	0.077	0.274	0.905
*R* ^2^	0.157	0.305	0.324	0.138	0.522	0.924
F value	2.624	6.172**	6.740*	2.252	6.671**	49.024***
max (VIF)	–	–	–	–	–	1.180
DH-test	5.894*	3.762	4.137	6.621*	5.274*	1.037
Observations	16					

Heteroskedasticity‒robustness (HAC) *t* statistics for standard errors are given in parentheses.

*** Significant at the 0.001 level (*p* < 0.001), **0.05 p-level, *0.1 p-level.

Table 5 describes the influence of club and training conditions on the number of certified riders. The information provided is derived from the perspectives of club coaches and owners. The regression coefficients indicate that an increase in the water surface available for aquatic training (0.353) and enhanced publicity for the sport (0.336) would result in a greater number of certified competitors.

**Table 5:** OLS regression model estimates based on Equation 3 (*N* = 16).

Dependent variable	COMP						
Explanatory variables	Model 1	Model 2	Model 3	Model 4	Model 5	Model 6	Model 7
Constant	5.399	6.128	5.009	4.323	3.354	3.646	5.038
	(12.71)***	(6.08)***	(6.80)***	(5.58)***	(4.27)***	(10.15)***	(3.43)***
CSUL	−0.413						−0.183
	(−0.41)**						(−2.21**
CSUA		−0.442					
		(−1.62)					
ETAM			−0.125				−0.242
			(−0.50)				(−2.53)**
ETLT				−0.039			
				(−0.14)			
ETWT					0.287		0.353
					(1.19)		(2.54)**
EPUB						0.336	
						(2.79)**	
Adj. *R*^2^	0.285	0.098	−0.052	−0.069	0.028	0.311	0.930
*R* ^2^	0.333	0.159	0.017	0.001	0.092	0.357	0.958
F value	7.005**	2.647	0.255	0.020	1.432	7.792**	34.632***
max (VIF)	–	–	–	–	–	–	2.452
DH-test	2.744	4.446	11.127***	10.053***	4.107	7.789**	5.123*
Observations	16						

Heteroskedasticity‒robustness (HAC) *t* statistics for standard errors are given in parentheses.

*** Significant at the 0.001 level (*p* < 0.001), **0.05 *p*-level, *0.1 *p*-level.

Conversely, the sentiments also indicate that superior conditions for training and preparation on land provided by sports facilities (−0.413) could diminish the number of competitors by increasing the amount of training provided. The evidence suggests that higher-quality training facilities discourage or create more favorable conditions, such as gyms being able to draw in potential competitors. The uneven distribution of state support and recognition in spectacular (Olympic) water sports might favor potential students who participate in swimming or water polo. The conditions provided by the sports facility regarding the quantity and quality of equipment available for aquatic and land-based training are not significant.

## 4 Discussions

Most swimming club coaches consider the improvement of training conditions to be of paramount importance, with a particular focus on enhancing training efficiency (18). Involves dealing with numerous logistical hurdles, such as integrating an advanced sound system, enabling easier removal of pitch ties, relocating the gymnasium away from the pool, and minimizing idle time. Additionally, there is a need to expand the water surface available for training, improve the quantity and quality of equipment, particularly for land-based training, and enhance the overall training environment.

However, there are also challenges and limitations associated with artistic swimming, including the need for ongoing support beyond the training circumstances. Furthermore, the necessity of training coaches and referees, the modernization of the scoring/judging systems, and the importance of teaching materials published by the Hungarian and international World Aquatics (earlier FINA) federations are important. The advantages of international training and hospitality abroad as a means of development, coupled with the staging of international-level competitions in domestic swimming pools, can enhance artistic swimming domestic recognition by attracting a larger audience than national-level championships.

In accordance with the present results, De Bosscher et al. (19) investigated the connection between elite sport policy and international success. This preliminary study included national sports policy questionnaires and input from athletes, coaches, and performance directors. Although the findings are not definitive, certain pillars such as financial resources, athlete support, training facilities, and coach development were highlighted in the most successful nations included in the sample (20). The available evidence indicates a nexus between economic development, higher level of funding and the success of national teams at the Olympic Games (21).

The emphasis on success in Hungary's elite sports and support for national sovereignty and identity can sometimes hinder the ability to gain support and accomplish goals. The necessity for significant financial investment in the organization of international sporting events places countries with limited resources at a disadvantage in terms of the level of public funding available for the development of their sporting infrastructure. One of the potential solutions to this issue is the involvement of private sponsors and companies (22).

Importantly, the financial stability of a club does not necessarily correlate with the success of its athletes. However, financial resources can indirectly increase their chances of success by providing better training conditions, higher-quality facilities, and a larger number of professionals and staff assisting their preparation. Furthermore, it enables the financing of coaching training, thereby enabling athletes to practice under the guidance of well-qualified professionals (23). These teams encompass professionals with diverse expertise of scientific and medical backgrounds, e.g., physiotherapists and psychologists (24), as well as crucial roles fulfilled by choreographers, ballet masters, and technicians (25).

Nevertheless, the provision of private funding poses the potential risk of the sponsoring entity unduly influencing the professionals previously responsible for the management of the club, thus potentially compromising the club's performance and standing (26). Furthermore, it has been proposed that community sports participation is associated with the financial burden on the athlete (family) and the necessity for adequate funding and infrastructure of sporting facilities. An inadequate training environment not only limits the number of potential professional competitors but also increases the risk of injury during training (27), and reduces enthusiasm for continued participation (28).

There are significant limitations to this study. When the results are analyzed, it is important to remain mindful of potential common method biases that could impact the accuracy and validity of the findings (29). These biases may have influenced participants’ responses, distorting the true nature of the data and potentially leading to overestimation or underestimation of certain effects, thereby affecting the conclusions drawn. This paper acknowledges the potential limitations of the OLS regression analysis as a statistical method, including overfitting and the assumption of linearity. Furthermore, it proposes alternative or complementary methodologies, such as the generalized linear model (GLM), to address these limitations and enhance the robustness of the regression analysis. A nonrepresentative dataset has the potential to misrepresent the true characteristics of a population, resulting in flawed interpretations and generalizations. In addressing this issue, it is advisable for forthcoming research to either employ a longitudinal study approach or undertake a comparative analysis with adjacent nations. The utilization of this approach would eradicate ambiguities regarding the data and illustrate the impact of business management tactics, the efficiency of subsidies and other resources on swimming sports over time. This could include more in-depth research into the economic factors influencing artistic swimming or an improved training environment for athletes’ performance.

Ultimately, the employment of suitably qualified professional coaches in each field of land-based and aquatic training is an effective technique for enhancing the well-being of athletes and the quality of training. This primary objective could be accomplished by implementing the necessary precautionary measures and specialized knowledge to increase training quality, minimizing the likelihood of injury and harm to athletes while maximizing training efficiency (30).

Another important practical application is that swimming clubs can prioritize the exploration of alternative funding sources, given that state funding, which is reliant on sponsorships, is an external factor that is beyond their control. To obtain the cooperation of private companies in the form of financial assistance, it is first necessary to generate interest in artistic swimming as a sport among the general public. One potential solution is the utilization of social media marketing, which has been demonstrated to be an effective method for generating engagement through the creation of content and advertisements tailored to the demographics of prospective new athletes.

## 5 Conclusions

This research illuminates the present state of training and sponsorship dynamics in Hungarian artistic swimming. By employing quantitative research, we were able to identify the necessary factors that have a significant effect on the certified competitors. Based on the proposed framework, we find that (1) the number of coaches and their qualifications positively affect the number of certified riders. (2) Different sources of funding, sponsorships in addition to government support, have positive effects, and membership fees have negative effects on the number of competitors. (3) An increase in the amount of water available for aquatic training and enhanced publicity for the sport would result in an increased number of certified competitors. However, better conditions for training and preparation on land provided by sports facilities could reduce the number of competitors.

This paper stresses the importance of physical well-being in artistic swimming and explores the ways in which land and aquatic training conditions can increase the participation. The findings are relevant to other Olympic sports, including rhythmic gymnastics, which demand a high degree of precision, coordination, and control (31). The artistry and aesthetics of swimming are similar to those of winter sports (e.g., skating), as both showcase athletes’ expression through routines and their embodiment of grace, fluidity and elegance (32).

Future research can investigate how differences in scientific training and expertise between countries affect the interpretation and applicability of study findings. The next step is to conduct a study across countries focusing on the economic barriers that could limit access to sports participation.

## Data Availability

The raw data supporting the conclusions of this article will be made available by the authors, without undue reservation.
